# A modified Tseng algorithm approach to restoring thoracic diseases’ computerized tomography images

**DOI:** 10.1371/journal.pone.0305728

**Published:** 2024-07-24

**Authors:** Dilber Uzun Ozsahin, Abubakar Adamu, Maryam Rabiu Aliyu, Huzaifa Umar

**Affiliations:** 1 Department of Medical Diagnostic Imaging, College of Health Science, University of Sharjah, Sharjah, UAE; 2 Research Institute for Medical and Health Sciences, University of Sharjah, Sharjah, UAE; 3 Operational Research Center in Healthcare, Near East University, Nicosia, Turkey; 4 Charles Chidume Mathematics Institute, African University of Science and Technology, Abuja, Nigeria; 5 Department of Energy System Engineering, Cyprus International University, Nicosia, Turkey; Bennett University, INDIA

## Abstract

It is well-known that the Tseng algorithm and its modifications have been successfully employed in approximating zeros of the sum of monotone operators. In this study, we restored various thoracic diseases’ computerized tomography (CT) images, which were degraded with a known blur function and additive noise, using a modified Tseng algorithm. The test images used in the study depict calcification of the Aorta, Subcutaneous Emphysema, Tortuous Aorta, Pneumomediastinum, and Pneumoperitoneum. Additionally, we employed well-known image restoration tools to enhance image quality and compared the quality of restored images with the originals. Finally, the study demonstrates the potential to advance monotone inclusion problem-solving, particularly in the field of medical image recovery.

## Introduction

Medical imaging is essential in the diagnosis and treatment of diseases as it provides direct guidance to medical personnel to cure diseases, and over the past decade, advancements in technology have brought about faster, more accurate, and less invasive medical devices [1]. Mathematical models in medical image restoration are a fundamental component of medical imaging, aiming to acquire high-quality images for clinical use while minimizing costs and risks to patients [2]. Intense data-driven models are highly flexible for extracting valuable information from massive data sets yet lack theoretical foundations [3]. Biomedical computing relies on mathematical models. Image data is fundamental in experimental, clinical, biomedical, and behavioural research [4]. Medical imaging problems, such as Magnetic Resonance Imaging (MRI), can be modelled as inverse problems. A modern and practical methodological approach, which has been widely applied, is based on the assumption that most real-life images have a low-dimensional nature. This method is highly effective and has proven to be successful [5]. The analysis of medical datasets through image processing techniques is a critical aspect of modern medical research. The development of algorithms for either partial or fully automatic analysis is essential in this context [6]. Image enhancement stands as a vital and complex method within the realm of image processing technology. Its fundamental goal is to improve the visual quality of an image or present a more polished representation of the picture.

Image restoration remains a critical area within medical image processing. It focuses on removing or reducing degradations in an image that may occur during the acquisition process. The ability to restore a medical image is essential for facilitating more accurate diagnosis and treatment [7]. Various types of medical images, including Computerized Tomography (CT) scans, Magnetic Resonance Imaging (MRI) scans, X-Ray images, microscopic images, and Ultrasound images, are prone to additive noise and blurring during the acquisition process [8–10]. Sources of image blurring may include optical distortions, motion during imaging, or atmospheric turbulence. Degradation of medical images can occur during transmission and acquisition, significantly impacting the analysis and processing of these images. Medical images with low spatial resolution and additive noise can lead to the misclassification of tumours or foreign objects, thus potentially compromising diagnosis and treatment outcomes [11]. Therefore, there is a need to explore additional evolutionary algorithms for addressing a wide range of medical imaging challenges [12]. The modified Tseng algorithms focus on removing image degradation that might occur during acquisition. Established methods such as deep learning are TV-based regularization approaches that effectively use gradient information to solve sparsity-constrained problems. It tends to improve the image quality of low-dose reconstructed images [13]. The key limitation associated with TV-based models lies in their tendency to encourage the recovery of images with sparse gradients. This characteristic can be beneficial for certain types of images but often results in an undesirable visual effect known as a “staircase”. CT scan images acquired are affected by ionizing radiations, which produce mottle noises that lead to the degradation of the images [14]. Similarly, such images can be restored using recent algorithms like Tseng. Multi-modality medical image fusion is a crucial technique in medical image processing. It is utilized extensively for diagnostic purposes with the aid of a co-occurrence filter and local extrema in a non-subsampled shearlet transform domain [15]. This approach integrates features from various imaging modalities, such as CT (Computed Tomography) and MRI (Magnetic Resonance Imaging), to create a new, composite medical image and enhance the information that clinicians can derive from the images [16].

Thoracic diseases (TD) pose significant health challenges, impacting a considerable number of individuals. Chest X-rays, widely utilized as a diagnostic method, play a crucial role in healthcare and are also referred to as computed tomography [17]. TD encompass a range of serious illnesses and health conditions, many of which exhibit a high prevalence. One illustrative example is pneumonia, which annually afflicts millions of individuals globally. In the United States alone, approximately 50,000 people succumb to pneumonia each year [18]. The chest X-ray (CXR) stands out as a widely used and cost-effective diagnostic instrument for identifying chest and thoracic diseases. Deciphering chest X-rays demands substantial expertise and careful visual scrutiny. Despite radiologists undergoing extensive clinical training and professional guidance, errors can still occur due to the intricate nature of diverse lung lesions and the subtle textural differences present in the images [19]. Precise chest X-ray (CXR) interpretations necessitate expert knowledge and medical experience. There have been extensive endeavours to automatically detect thoracic diseases (TD) using CXR data. However, increasing image volumes and subtle texture changes can lead to errors, even by experienced radiologists [20]. Statistical learning methods, such as Support-vector networks [21, 22], Bayesian classifiers [23, 24], and k-nearest neighbour algorithms [19], are not adept at directly handling high-dimensional pixel-level features in medical images. The patterns of diseases found in chest X-rays are numerous, and their occurrence follows a long-tailed (LT) distribution [25]. Therefore, it is essential to have an efficient and reliable mathematical algorithm that can restore the images and easily detect different types of diseases.

The image restoration can be achieved in various forms, such as image denoising [21], deblurring [26], inpainting’ [27, 28], dehazing [29], and de-raining [30]. Numerical simulations should be the best option in solving problems related to medical imaging analysis, as factors such as specular noise can affect the interpretation of the image obtained. Therefore, complex mathematical models using various algorithms are needed to study medical images. In this study, various thoracic diseases computerized tomography (CT) images degraded with known blur function and additive noise were restored using a modified Tseng algorithm. Furthermore, we utilized well-known image restoration tools to enhance image quality and compare the quality of the restored images with the original images.

## Methodology

The thoracic disease patients’ CT scan images were collected from an archive of medical images of cancer (https://www.cancerimagingarchive.net); they represent various disease conditions, and ethical approval is not applicable [26].

**Fig 1:**
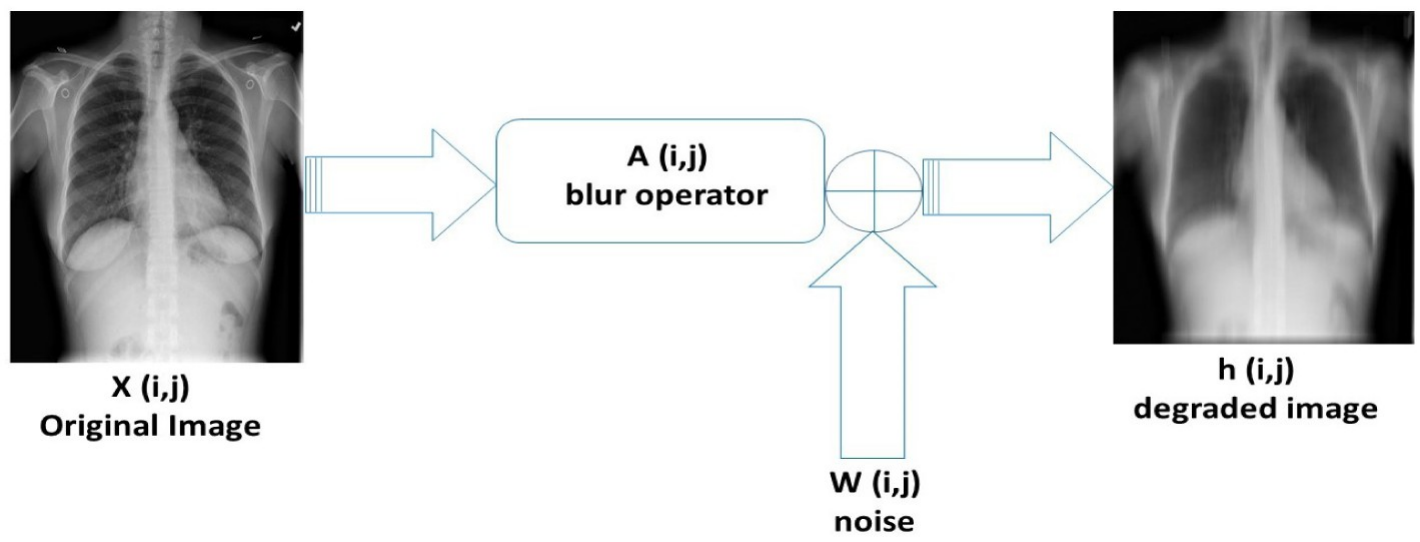
Image degradation.

Mathematical models used for image restoration problems are often formulated using Eq (1), and illustrated in Fig 1:

$$\begin{array}{c} y=Dx+\eta , \end{array}$$ (1)

where *y* is the observed image, *D* is the degradation function, *x* is the original image and *η* is noise.

The objective of this study is to restore a degraded image as illustrated in Fig 1 using mathematical algorithms. Since the solution may not be unique for any degraded image, this problem inherits ill-posedness. To restore well-posedness, regularization techniques are employed. The *l*_1_ regularization method is known to be a powerful technique for image denoising and deblurring problem. The formulation is given by

$$\begin{array}{c} \underset{x}{\text{arg}\;\text{min}}\frac{1}{2}{\left\|Dx-\eta \right\|}^{2}+\mu {\left\|x\right\|}_{1}, \end{array}$$ (2)

where *μ* is the regularizing term. By simple mathematical reformulation, solutions of the minimization problem (2) are equivalent to solutions of the inclusion problem:

$$\begin{array}{c} \text{find}\;\;u\in H\;\text{such}\;\text{that}\;\;\;0\in (\nabla f(u)+\partial g(u)), \end{array}$$ (3)

where *H* is a real Hilbert space, ∇*f* is the gradient of *f* and ∂*g* is the subdifferential of *g*, with

$$f\left(x\right)≔\frac{1}{2}{\left\|Dx-\eta \right\|}^{2}\;\;\;\text{and}\;\;\;g\left(x\right)≔{\left\|x\right\|}_{1}.$$

Then,

$$\nabla f\left(x\right)=D^{T}\left(Dx-\eta \right)\;\;\;\text{and}\;\;\;\partial g\left(x\right)=\{\begin{array}{c} \frac{x}{\left\|x\right\|},\;x\neq 0;\\ \{x:\|x\|\leq 1\},\;x=0. \end{array}$$

In the literature, several algorithms introduced for approximating zeros of sum of two monotone operators have been used to solve the inclusion problem (3) (see, e.g., [31–40] and the references therein). We propose a modification of the popular Tseng algorithm for approximating solutions of problem (3). Our propose method is the following:

**Algorithm 1 Step 1**. *Given x*_0_ = *Dx* + *η*, λ = 0.001, *μ* = 0.3 *and set k* = 1.

**Step 2**. *Compute w*_*k*_ *and x*_*k*+1_,

$$\begin{array}{c} \left\{ \begin{array}{c} w_{k}={\left(I+\lambda \mu \partial g\right)}^{-1}\left(x_{k}-\lambda \nabla f\left(x_{k}\right)\right),\\ x_{k+1}=w_{k}-\lambda \left(\nabla f\left(w_{k}\right)-\nabla f\left(x_{k}\right)\right), \end{array}\right. \end{array}$$ (4)

*where I is the identity mapping*.

**Step 3** *Set k* ← *k* + 1, *and* **go to Step 2**.

### Convergence analysis

**Theorem 2** *The sequence* {*x*_*k*_} *generated by our proposed method converges to a solution of problem 2*.

**Proof**. Since ∇*f* is Lipschitz and ∂*g* is monotone, the proposed method can be viewed as a corollary of the famous Tseng algorithm [36]. Hence, the convergence analysis follows using a similar argument given in [36].

## Experimental results and discussion

**Fig 2:**
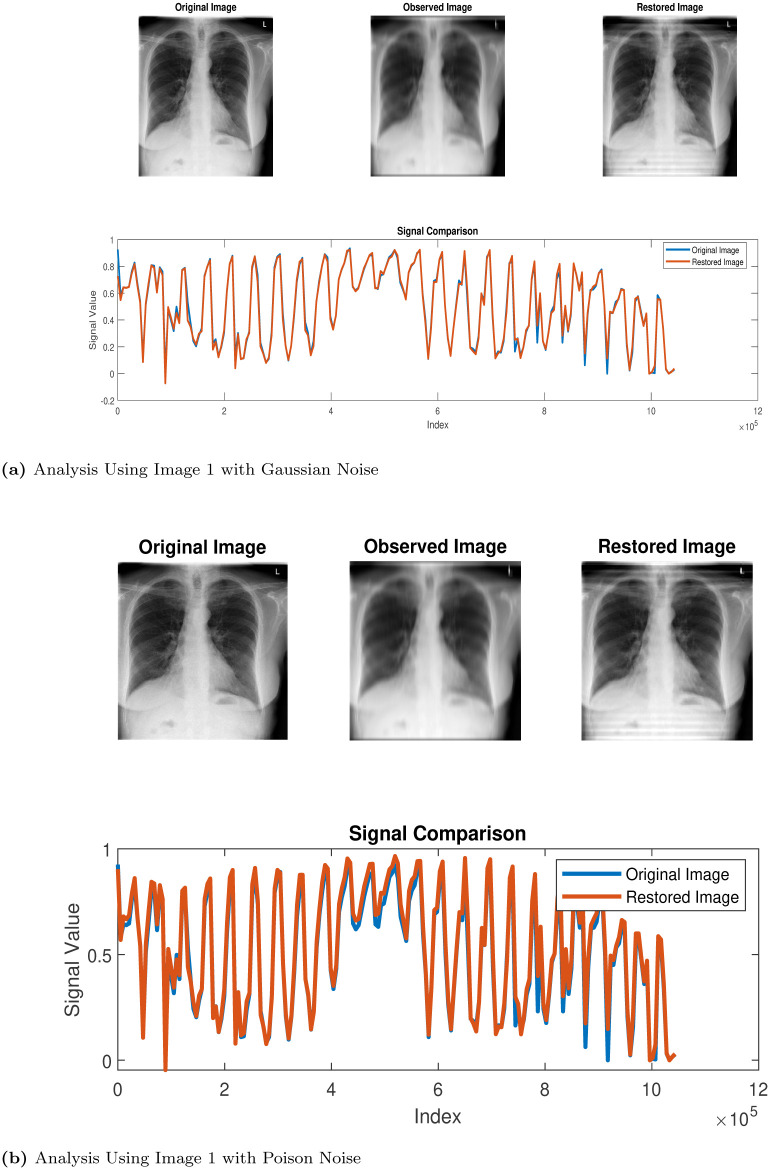
Restoration process via Algorithm 1. (a) Analysis Using Image 1 with Gaussian Noise, (b) Analysis Using Image 1 with Poison Noise.

**Fig 3:**
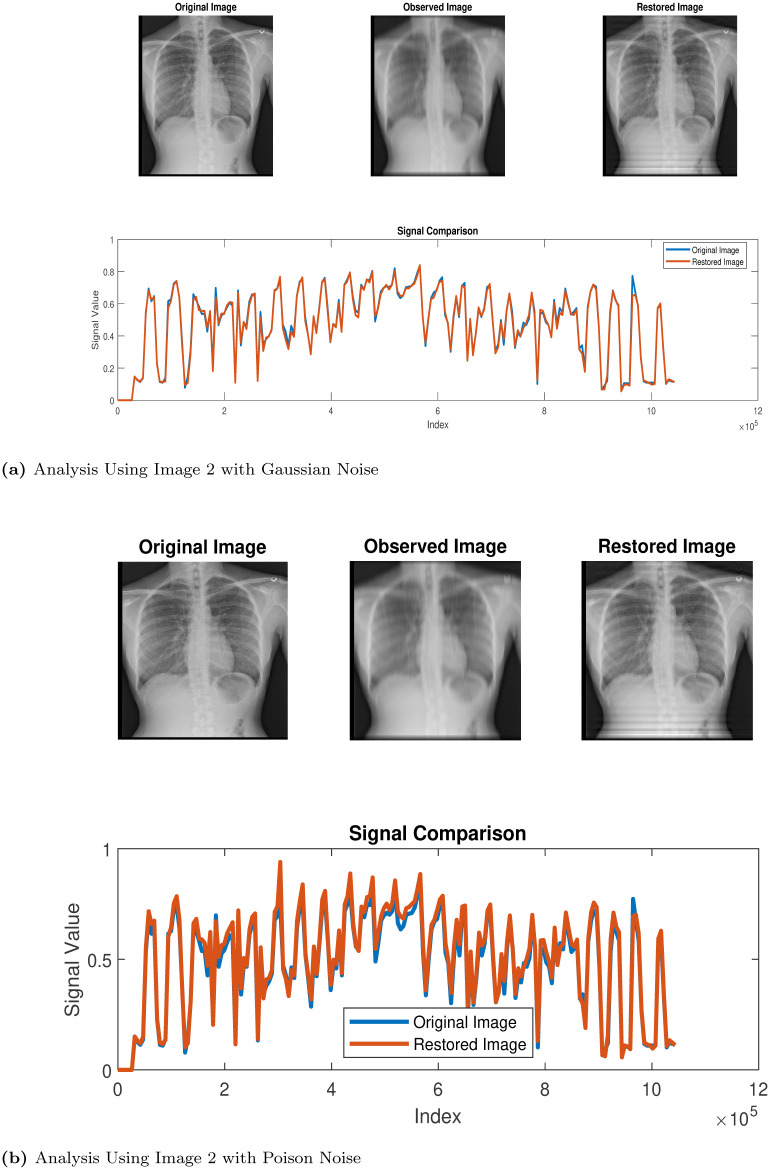
Restoration process via Algorithm 1. (a) Analysis Using Image 2 with Gaussian Noise, (b) Analysis Using Image 2 with Poison Noise.

**Fig 4:**
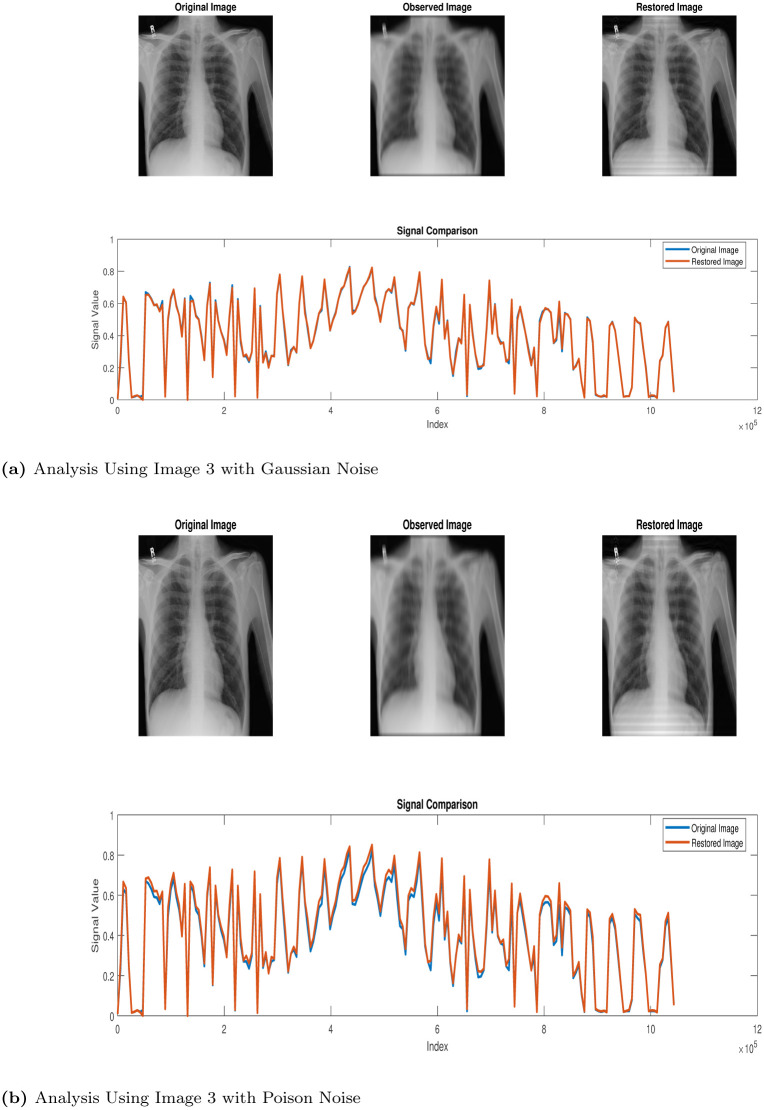
Restoration process via Algorithm 1. (a) Analysis Using Image 3 with Gaussian Noise, (b) Analysis Using Image 3 with Poison Noise.

**Fig 5:**
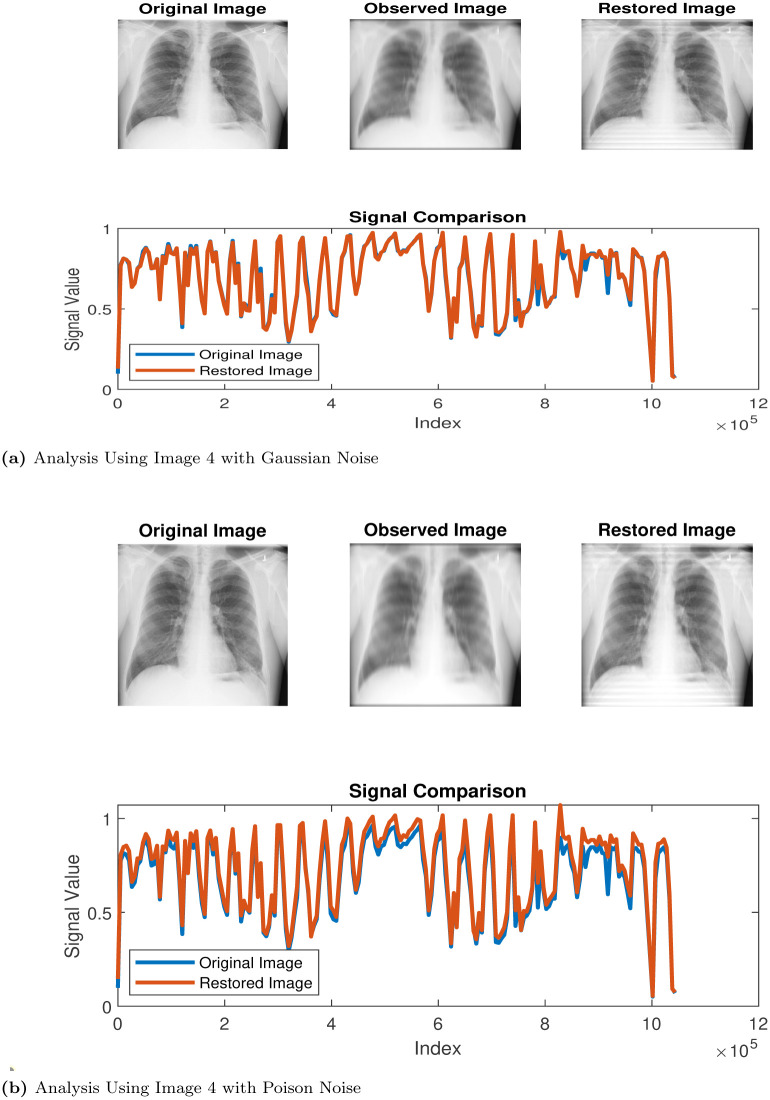
Restoration process via Algorithm 1. (a) Analysis Using Image 4 with Gaussian Noise, (b) Analysis Using Image 4 with Poison Noise.

**Fig 6:**
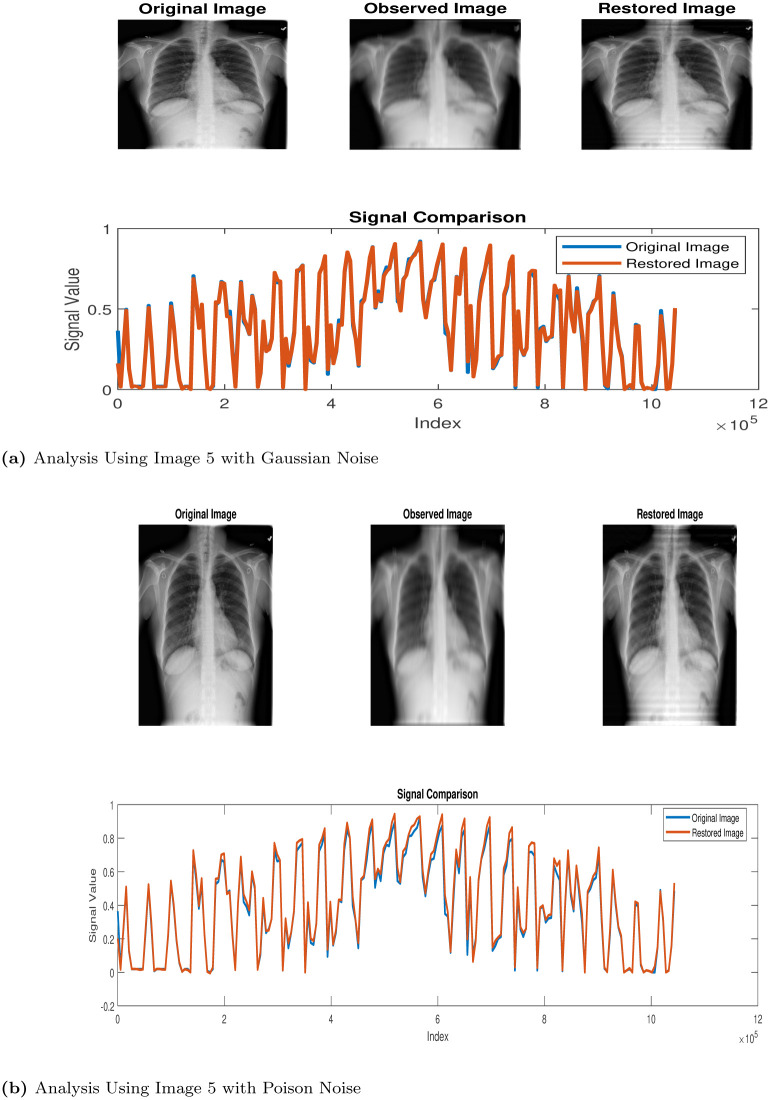
Restoration process via Algorithm 1. (a) Analysis Using Image 5 with Gaussian Noise, (b) Analysis Using Image 5 with Poison Noise.

In this section, we use the proposed method (Algorithm 1) in the restoration process of some CT scan images obtained from thoracic disease patients [26] We label the images as follows: Images 1, 2, 3, 4 and 5 represent Calcification of the Aorta, Subcutaneous Emphysema, Tortuous Aorta, Pneumomediastinum and Pneumoperitoneum, respectively. We study the behaviour and properties of the restored images when they are degraded using MATLAB’s built-in motion blur function (*P* = *fspecial*(′*motion*′, 30, 60)), and we added Gaussian noise (GN) and poison noise (PN) with scaling factor *σ* = 0.001 and 0.05, respectively. The results of the simulations are presented in the Figs 2–6 below:

### Discussion

Our algorithm was applied to restore computerized tomography (CT) images depicting various thoracic diseases. These images had been intentionally degraded with known blur and additive noise. The implementation of our algorithm led to an enhanced restoration performance for the degraded images. Despite this, the models we introduce exhibit effectiveness specifically in the areas of deblurring and denoising operations, ultimately yielding accurate restoration results.

Clearly, looking at the images in Figs 2–6, one can easily see that the proposed Algorithm 1 restored the test images effectively. However, to validate this claim, there are tools used for measuring the quality of restored images. We will use three (3) different metrics to analyze the qualities of the restored images. These tools are, namely, the structural dissimilarity index measure (SSIM), the Improvement in signal-to-noise ratio (ISNR) and the signal-to-noise ratio (SNR) index. These metrics are expressed, respectively, as follows:

$$\begin{array}{c} \text{SSIM}\left(x,y\right)=\frac{\left(2\mu_{x}\mu_{y}+c_{1}\right)\left(2\sigma_{xy}+c_{2}\right)}{\left(\mu_{x}^{2}+\mu_{y}^{2}+c_{1}\right)\left(\sigma_{x}^{2}+\sigma_{y}^{2}+c_{2}\right)}, \end{array}$$ (5)

where *x* and *y* represent the original and restored images, *μ*_*x*_ and *μ*_*y*_ denote the mean values of *x* and *y*, *σ*_*x*_ and *σ*_*y*_ are the standard deviations of *x* and *y*, *σ*_*xy*_ is the covariance between *x* and *y*, and *c*_1_ and *c*_2_ are small constants introduced to prevent division by zero.

$$\begin{array}{c} \text{ISNR}≔10\;\text{log}\frac{{\left\|x-y\right\|}^{2}}{\|x-x_{n}\|},\;\text{and}\;\text{SNR}≔10\;\text{log}\frac{\left\|x\right\|}{\|x-x_{n}{\|}^{2}}, \end{array}$$ (6)

where *x*, *y*, and *x*_*n*_ denote the original, observed, and estimated images at iteration *n*, respectively.

The SSIM value ranges from 0 to 1, with 1 denoting perfect recovery while higher values for SNR and ISNR indicate superior restoration. The performance of our proposed Algorithm 1 using these metrics is detailed in Table 1 below.

**Table 1:** SSIM, ISNR and SNR for the test images.

	Algorithm 1 (GN)				Algorithm 1 (PN)			
Test image	SSIM	ISNR	SNR	Time	SSIM	ISNR	SNR	Time
Image 1	0.89	7.67	61.19	24.36	0.89	4.30	53.40	26.31
Image 2	0.92	4.72	59.96	26.21	0.92	2.36	52.93	24.45
Image 3	0.94	7.05	66.48	25.36	0.95	2.92	55.73	24.02
Image 4	0.93	7.26	68.58	27.89	0.94	2.72	56.32	25.97
Image 5	0.91	6.92	62.01	26.28	0.91	3.66	53.94	25.70

**Remark 1** *From the metrics of the restored images presented in* Table 1, *it is convincing that our proposed Algorithm 1 restored the test images with high quality*.

## Conclusion

Overall, this study successfully restored computerized tomography (CT) images of various thoracic diseases that had been degraded with known blur and additive noise. The restoration was achieved through the implementation of a mathematical algorithm, specifically a modified Tseng algorithm. Furthermore, we employed established image restoration tools to enhance the quality of the images and conducted a comprehensive comparison between the restored images and the original ones. This approach not only showcases the efficacy of the applied algorithm but also underscores the importance of combining mathematical models with established tools in the field of medical image restoration.
